# Successful Perioperative Combination of High-Dose FVIII Therapy Followed by Emicizumab in a Patient with Hemophilia A with Inhibitors

**DOI:** 10.1055/s-0039-3401001

**Published:** 2019-12-05

**Authors:** Shuichi Okamoto, Nobuaki Suzuki, Atsuo Suzuki, Sachiko Suzuki, Shogo Tamura, Mochihito Suzuki, Nobunori Takahashi, Toshihisa Kojima, Takeshi Kanematsu, Tetsuhito Kojima, Hitoshi Kiyoi, Naoki Ishiguro, Tadashi Matsushita

**Affiliations:** 1 Department of Hematology and Oncology, Nagoya University Graduate School of Medicine, Nagoya, Japan; 2 Department of Transfusion Medicine, Nagoya University Hospital, Nagoya, Japan; 3 Department of Medical Technique, Nagoya University Hospital, Nagoya, Japan; 4 Department of Pathophysiological Laboratory Sciences, Nagoya University Graduate School of Medicine, Nagoya, Japan; 5 Department of Nursing and Health, Aichi Prefectural University, Aichi, Japan; 6 Department of Orthopaedics/Rheumatology, Nagoya University Graduate School of Medicine, Nagoya, Japan; 7 Department of Clinical Laboratory, Nagoya University Hospital, Nagoya, Japan

**Keywords:** factor VIII inhibitors, surgery, hemophilia therapy

## Abstract

We managed perioperative hemostasis for a 72-year-old man with hemophilia A and low inhibitor titers (3 BU/mL), who underwent osteosynthesis for supracondylar fracture of the left humerus. He was treated perioperatively using the combination of high doses of factor VIII (FVIII) with recombinant human Factor VIII Fc fusion protein (rFVIIIFc), followed by emicizumab. On the day of surgery (day 0), he was administered bolus infusion of 150 IU/kg rFVIIIFc, followed by continuous infusion at a dose of 4 IU/kg/h. Emicizumab, 3 mg/kg, was injected subcutaneously once a week, on days 5, 12, 19, and 26. Inhibitors were detected on day 6 at a titer of 4 BU/mL and FVIII:C decreased to below assay sensitivity limits on day 10. The rate of increase in inhibitor titers was high, with inhibitors increasing to 343.4 BU/mL on day 14. The transition of thrombin production by thrombin generation assay (TGA) showed temporary decrease in thrombin production on day 7, although it was restored by day 10, i.e., five days after commencement of emicizumab therapy. Rotational thromboelastometry displayed consistent results with TGA, showing that clotting time was prolonged and the alpha angle decreased to less than measurable levels on day 6, although they were improved by day 10. There were no bleeding-related events or other adverse events throughout the perioperative period. In conclusion, emicizumab was effective for the management of perioperative hemostasis after development of an anamnestic response in a patient with hemophilia A with inhibitors. Combination therapy with high doses of FVIII followed by emicizumab could be a workable alternative for patients with hemophilia A with inhibitors.

## Introduction

Bypassing therapy using bypassing agents and/or administration of high doses of factor VIII (FVIII) products have been used to manage severe bleeding or perioperative hemostasis in hemophilia A patients with inhibitors. 1 2 3 Generally, high-dose FVIII administration is preferred over bypassing therapy for patients with low titers of inhibitors, because the hemostatic effects are more stable than those with bypassing therapy. 4 However, an anamnestic response that develops several days after high-dose FVIII administration makes continuation of this therapy difficult. Hence, the combination of high-dose FVIII therapy followed by bypassing agents has been employed during the perioperative period of major surgery, although the timing for changing from high-dose FVIII administration to bypassing therapy is difficult to judge.

Emicizumab is a new agent for the prevention of bleeding in hemophilia A patients with inhibitors. This humanized bispecific antibody binds FIXa and FX, acting as a substitute for the hemostatic effects of FVIII products. 5 However, whether or not emicizumab can be used in the perioperative management of hemophilia A patients has not been elucidated. We describe a hemophilia A case in which we used emicizumab in combination with high-dose FVIII therapy in the perioperative period.

## Case Presentation

We managed perioperative hemostasis for a 72-year-old man with hemophilia A and low inhibitor titers (3 BU/mL), as estimated using Bethesda assay, who underwent osteosynthesis for supracondylar fracture of the left humerus. He was treated perioperatively using the combination of high doses of FVIII with recombinant human FVIII Fc fusion protein (rFVIIIFc), followed by emicizumab.

**Fig. 1:**
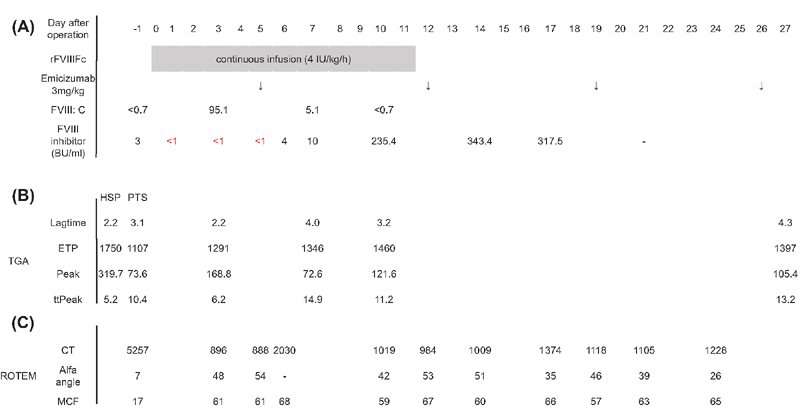
The patient's clinical course and results of thrombin generation assay (TGA) and rotational thromboelastometry (ROTEM). Chromogenic substrate assay for FVIII:C and the Bethesda assay for FVIII inhibitors were modified to avoid the influence of emicizumab. 7 Inhibitors were below measurement sensitivity on days 1, 3, and 5 and were detected on day 6, at a titer of 4 BU/mL. FVIII:C decreased to below assay sensitivity limits on day 10, in spite of continuous infusion of rFVIIIFc. Emicizumab was first administered on day 5 at a dose of 3 mg/kg, this dose being administered four times at weekly intervals, followed by 1.5 mg/kg weekly. ( **A** ) TGA: TGA was performed using citrated platelet-poor plasma (PPP) from the patient, extracted using a PPP reagent. The procedure was performed using calibrated automated thrombography (Thrombinoscope BV; Finggal link, Tokyo, Japan), in accordance with the manufacturer's instructions. We monitored reactions for 1 hour, using a Fluoroskan Ascent FL microplate fluorometer (Thermo Fisher Scientific, Tokyo, Japan), set at an excitation wavelength of 390 nm and an emission wavelength of 460 nm, and Thrombinoscope software (Thrombinoscope BV). The wave shapes of TGA and an explanation of the parameters are shown in Supplementary Fig. S1 . ( **B** ) ROTEM: The NATEM mode was used in this study, which is reportedly more informative than EXTEM and INTEM modes in patients being treated with emicizumab. 8 Blood samples were collected in a tube containing 3.2% trisodium citrate. Whole blood (300 µL) was mixed with 20 µL of star-TEM (CaCl _2_ ; final concentration, 12.5 mM), then analyzed using a whole-blood hemostasis analyzer. The coagulation process was assessed using clotting time (CT; time from start of measurement until detection of clot firmrmness at 2-mm amplitude), α angle (angle of the tangent between 0 mm and the curve when the clot firmness is 20 mm), and maximum clot firmness (MCF, firmness of the clot and clot quality) ( **C** ). CT, clotting time; ETP, endogenous thrombin potential; HSP, human standard plasma; MCF, maximum clot firmness; PTS, pretreatment state; rFVIIIFc, recombinant human factor VIII Fc fusion protein; ROTEM, rotational thromboelastometry; TGA, thrombin generation assay; ttPeak, time to peak.

The patient's clinical course is shown in Fig. 1 . On the day of surgery (day 0), he was administered bolus infusion of 150 IU/kg rFVIIIFc, followed by continuous infusion at a dose of 4 IU/kg/h. Emicizumab, 3 mg/kg, was injected subcutaneously once a week, on days 5, 12, 19, and 26 ( Fig. 1A ).

*Transition of FVIII:C and inhibitors* : Inhibitors were detected on day 6 at a titer of 4 BU/mL and FVIII:C decreased to below assay sensitivity limits on day 10 ( Fig. 1A ). The rate of increase in inhibitor titers was high, with inhibitors increasing to 343.4 BU/ml on day 14. These results indicate that the effect of high-dose FVIII administration was completely negated by inhibitors by day 10, although the continuous infusion of rFVIIIFc was continued up to day 11. This suggests that the coagulation and hemostatic effects solely depended on emicizumab on and after day 10.

*Assessment by thrombin generation assay (TGA)* ( Fig. 1B ): On day 3, FVIII:C levels were 95.1% and inhibitor titers were less than assay sensitivity limits with high-dose FVIII therapy. Lag time, peak, and time to peak were significantly improved compared with the pretreatment state. In particular, lag time was equal to that of human standard plasma. On day 7, all parameters except for endogenous thrombin potential worsened, with lag time and time to peak being even longer than in the pretreatment state. On days 10 and 27, the peak significantly improved again, although lag time and time to peak were comparable to those on day 7.

*Assessment by rotational thromboelastometry (ROTEM)* ( Fig. 1C ): All parameters were significantly improved by administration of high doses of FVIII on days 3 and 5 compared with the pretreatment state. Although clotting time was prolonged and the α angle decreased to less than measurable levels on day 6, these parameters were significantly improved on day 10. Further, the data on and after day 21 were almost comparable to those of day 10, with only a decrease in the α angle on day 24.

There were no adverse events and symptoms suggesting worsening of hemostatic effects, such as decrease in hemoglobin levels, throughout the perioperative period. Hence, blood transfusions and additional treatment with bypassing agents were not required.

## Discussion

The transition of FVIII:C and FVIII inhibitors in this case indicated that high-dose FVIII administration became ineffective between days 6 and 10. The results of coagulation tests, TGA and ROTEM, supported these observations, indicating worsening of coagulation on days 6 and 7, which might have been due to an anamnestic response and failure of high-dose FVIII administration. However, since there was no worsening of hemostasis clinically, additional infusion of bypassing agents was not required. Further, these results improved on day 10, probably as a response to emicizumab, because the effect of high doses of FVIII seemed to be weak, if any, due to the high titers of inhibitor.

In terms of pharmacokinetics, emicizumab reportedly requires about 4 weeks to reach a steady state with weekly infusions. 6 However, no reports have elucidated the relationship between emicizumab concentration and coagulation effects. Additionally, in our patient, all coagulation test parameters on and after day 10 were broadly comparable. This indicates that the coagulation effect of emicizumab had almost reached a steady state by day 10, 5 days after commencing emicizumab therapy. These results suggest that if emicizumab had been started earlier, for example, on day 2 or so, worsening of the coagulation effect by the anamnestic response could have been avoided. Further, if the commencement period of emicizumab is precisely optimized, the combination of high-dose FVIII therapy followed by emicizumab might provide a workable alternative for patients with hemophilia A with inhibitors, thus eliminating the need to juggle the timing of changing the therapy from high-dose FVIII to bypassing agents.

However, there is considerable doubt whether emicizumab can be used in the management of major surgery as a substitute for high-dose FVIII therapy, because in a comparison of the results of coagulation tests between high-dose FVIII administration and emicizumab, high doses of FVIII displayed more favorable results for most parameters, indicating that high-dose FVIII therapy can produce more stable coagulation effects than emicizumab.

In conclusion, emicizumab was effective for the management of perioperative hemostasis after development of an anamnestic response in a patient with hemophilia A with inhibitors. Combination therapy with high doses of FVIII followed by emicizumab could be a workable alternative for patients with hemophilia A with inhibitors.
