# Supplementary material for: Successful Perioperative Combination of High-Dose FVIII Therapy Followed by Emicizumab in a Patient with Hemophilia A with Inhibitors
Source: TH Open. 2019 Dec 5;3(4):e364–6. doi: 10.1055/s-0039-3401001 (PMC6894946; doi:10.1055/s-0039-3401001)
Supplement: Supplementary file 1 — Supplementary Material [file 10-1055-s-0039-3401001-s190044cr.pdf]

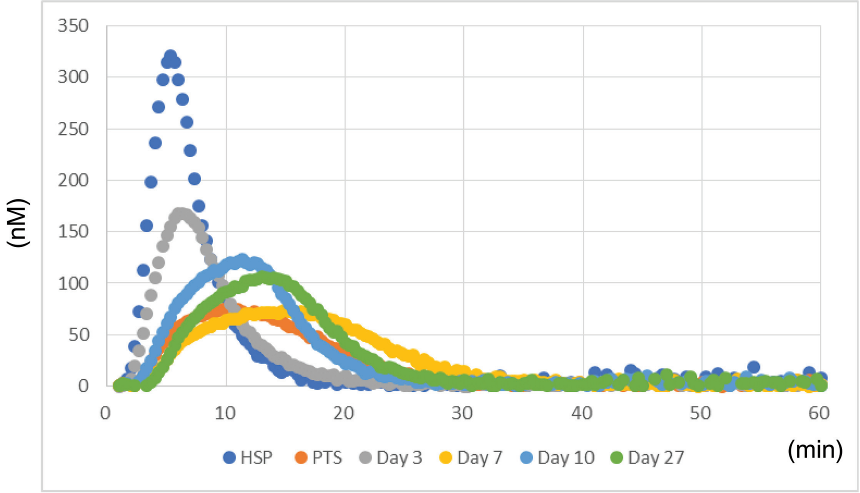

Supplementary Fig. S1 Wave shapes of thrombin generation assay. Lag time is the time from start of the reaction to initiation of thrombin generation. Endogenous thrombin potential is the area under the curve, peak is the highest thrombin level, and time to peak (ttPeak) is the time from start of the reaction to the peak.[8] HSP, human standard plasma; PTS, pretreatment state.
